# 3D mixed-reality visualization of medical imaging data as a supporting tool for innovative, minimally invasive surgery for gastrointestinal tumors and systemic treatment as a new path in personalized treatment of advanced cancer diseases

**DOI:** 10.1007/s00432-021-03680-w

**Published:** 2021-06-10

**Authors:** Ryszard Wierzbicki, Maria Pawłowicz, Józefa Job, Robert Balawender, Wojciech Kostarczyk, Maciej Stanuch, Krzysztof Janc, Andrzej Skalski

**Affiliations:** 1 Department of Surgical Oncology, NEO HOSPITAL Sp. z o. o. ONE Sp. k., Ul. Józefa Kostrzewskiego 47, 30-437 Kraków, Poland; 2 Department of Clinical Oncology, NEO HOSPITAL Sp. z o. o. ONE Sp. k., Ul. Józefa Kostrzewskiego 47, 30-437 Kraków, Poland; 3 Department of Surgical General, NEO HOSPITAL Sp. z o. o. ONE Sp. k., Ul. Józefa Kostrzewskiego 47, 30-437 Kraków, Poland; 4 Department of Measurement and Electronics, AGH University of Science and Technology, al. Mickiewicza 30, Kraków, Poland; 5 MedApp S.A., al. plk. Beliny-Prazmowskiego 60, Kraków, Poland

**Keywords:** Imaging, Three-dimensional, Ablation techniques, Irreversible electroporation, Personalized medicine, Cancer

## Abstract

**Background:**

The purpose of this study was to investigate the potential of a combination of 3D mixed-reality visualization of medical images using CarnaLife Holo (MedApp, Poland) system as a supporting tool for innovative, minimally invasive surgery/irreversible electroporation—IRA, Nano-Knife), microwave ablation (MWA)/for advanced gastrointestinal tumors. Eight liver and pancreatic tumor treatments were performed. In all of the patients undergoing laparoscopy or open surgery volume and margin were estimated by preoperative visualization. In all patients, neoplastic lesions were considered unresectable by standard methods.

**Methods:**

Preoperative CT or MRI were transformed into holograms and displayed thanks to the HoloLens 2. During operation, the surgeon’s field of view was augmented with a 3D model of the patient’s relevant structures.

**Results:**

The intraoperative hologram contributed to better presentation of tumor size and locations, more precise setting of needles used to irreversible electroporation and for determining ablation line in case of liver metastases. Surgeons could easily compare the real patient's anatomy to holographic visualization just before the operations.

**Conclusions:**

The combination of 3D mixed-reality visualization using CarnaLife Holo with IRA, MWA and next systemic treatment (chemotherapy) might be a new way in personalized treatment of advanced cancers.

## Introduction

The fields of 3D image-guided interventional therapies are rapidly growing, both in technical development and clinical adoption. Most cancerous tumors are now diagnosed with computed tomography (CT), magnetic resonance imaging (MRI), or ultrasound imaging (USG). Surgical treatment continues to be the preferred treatment for most solid tumors and crucial for the prognosis. However, for many cancer patients there is no chance for surgical removal due to difficult-to-reach or unresectable neoplasm. This situation puts pressure on the multidisciplinary teams to find alternative methods of treatment that achieve not only a response rate but also prolong a patient’s lifespan and will improve the quality of life. Classical methods of open surgery are usually traumatic, require long-lasting general anesthesia, several days of hospital and outpatient’s recovery before the patient can start chemotherapy, which is an essential component of the treatment of advanced cancers.

In the case of the pancreatic tumor situated near the hepatic artery, celiac trunk, superior mesenteric vessels, a neoplastic infiltration of the above-mentioned structures contributes to surgical inaccessibility. Thermal ablation is associated with high morbidity due to the presence of these fragile structures. Thus, IRE plays a critical role in the management of patients with locally advanced disease.

The liver is frequently a site of metastasis, especially for primary tumors of the gastrointestinal tract. Performed with a 10-mm safety margin, MWA offers very good oncological results and low morbidity and is considered an option not only in high-risk patients but also as an alternative to very aggressive surgery in selected cases.

Thermal ablation is often not an option due to the presence of tumors on or near hepatic blood vessels or biliary tract, and patients with underlying liver dysfunction have increased rates of post-treatment abscess formation after thermal ablation. IRE due to its nonthermal nature allows to overcome many of these limitations and has shown to be suitable for ablating tumors near vital hepatic structures.

Notably, Bhutiani et al. (2016) found that IRE had a similar 6-month success rate but was more tolerable than microwave ablation for patients with compromised liver function (Child–Pugh B) IRE-treated patients also had shorter hospital stays and lower rates of re-admission, likely due to lower indiscriminate effects on hepatic tissue.

Mixed Reality was firstly tried in 1968 by Ivan Sutherland who created the head-mounted display called The Sword of Damocles (Fuchs and Ackerman 1999). The development of this field was slow due to technology limitations. In 2016 Microsoft released the HoloLens HMD which enabled the first tasks including mixed reality to be carried out in medicine. Since then, the technology has developed from the concept to a product that is available in the operational theaters (Tepper et al. 2017). The mixed reality can be used in many fields of medicine like open surgery (Fida et al. 2018), orthopedic surgery (Verhey, 2020), healthcare education (Gerup et al. 2020; Thomas et al. 2006) or rehabilitation (Duff et al. 2010). There were also cases for use of the mixed reality with the real-time streaming of the imaging data from echocardiography in laparoscopic procedures (Kasprzak et al. 2019a, b). In 2020 HoloLens 2 was released which gives now more features including better ergonomics, precise hand gesture recognition and higher visualization quality.

Presentation of 3D structures in mixed reality gives a better perception of the depth in the visualized structures. This results in the quicker and more accurate localization of the pathological lesions in the imaging data. A test was conducted on 28 clinicians with varying medical experience. They had to identify liver lesions using the classical approach and using mixed reality. It turned out that the median time for correct identification was 23.5 (4–138) s using the classic method and 6.00 (1–35) s using HoloLens (*p* < 0.001) (Pelanis^ 2019^).

Intraoperative hologram support was already tried for liver surgery operations but on a limited number of patients (Saito et al. 2020). The results of the works are proving that thanks to the use of HoloLens the surgeon achieved better spatial awareness and could interact with the visualization without breaking the sterile field.

This article presents a combination of engineering—3D mixed reality/MR/techniques of medical data imaging using CarnaLifeHolo system, that is using the newest head-mounted display—Microsoft HoloLens 2, as a supporting tool for an innovative, minimally invasive surgery (MIS)/irreversible electroporation—IRA), microwave ablation (MWA)/as a therapeutic option for patients disqualified from surgical treatment due to neoplastic lesions considered to be unresectable.

## Methods

### Population

8 patients diagnosed with liver and pancreatic tumors were included in our study. They were treated in Neo Hospital, Cracow, Poland in the timeframe between 16th June 2020 and 30th October 2020. All of the patients had diagnostic imaging data (CT/MRI) that were used later on for treatment planning and support. The patients were diagnosed with primary tumors and metastases in the area of the abdominal cavity. 2 patients with metastatic colorectal cancer (mCRC), 2 patients with pancreatic cancer—1 locally advanced and 1 with synchronous liver metastases, 2 with metachronous liver metastases from breast cancer, 2 with extrahepatic cholangiocarcinoma. 4 female patients and 4 male patients. The mean age was 62 years with the standard deviation (SD) equal to 10.35. CT was the preferred imaging technique for diagnosis and optimal tumor assessment. The Medical Research Involving Human Subjects Act did not apply to this study. All patients were asked for informed consent. All of the patients' relevant information is summed up in Table 1.

**Table 1:** Patients characteristics

Patient no./sex/age	Location of primary tumor/metastatic site	Previous therapy	Preoperative conventional imaging available	Surgical procedures performed
1. M/65	Bile duct/Hepar (hilum, right and left lobe)/	No	CT/MRI	Laparotomy, IR, MWA
2. F/40	Pancreas/Hepar (VI, V segment)	No	CT/MRI	Laparotomy, IRE, MWA
3. M/72	Pancreas	Surgery	CT	Laparotomy, IRE
4. M/62	Colon/Hepar (VII/VIII,VI segment)	Surgery, percutaneous thermal ablation, chemotherapy	CT	Laparotomy, IRE, MWA
5. F/56	Breast/Hepar (VII/VIII, II/III segment)	Surgery, chemotherapy	CT	Laparoscopy, MWA
6. F/46	Colon/Hepar (VIII segment)	Surgery, chemotherapy	CT	Laparotomy, IRE
7. F/66	Breast/Hepar (VI, VI/VII)	Surgery/chemotherapy	CT/MRI	Laparotomy, MWA
8. M/72	Hepar	No	CT/MRI	Laparotomy, IRE

Candidates for IRE were qualified by an oncologist surgeon, on the basis of preprocedural imaging/CT, MRI/, which were transformed into 3D holograms, performance status [documented using the Eastern Cooperative Oncology Group (ECOG) criteria (Oken et al. 1982)], a serum blood count, coagulation tests, renal function, metabolic panel, and detailed cardiac history.

**Fig. 1:**
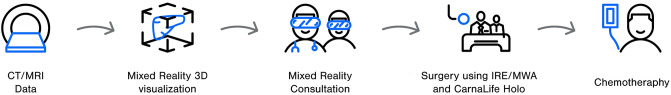
Workflow of the combination of mixed reality visualization and surgery

In seven cases the abdominal cavity was opened using a diathermy knife (system Soft) creating a clean incision and bloodless field to operate. In one of the cases, the procedure was performed using the laparoscopic method. In all of the cases, the CarnaLife Holo mixed reality visualization was used for pre-operative planning and also in the operating room to support decision-making process concerning the further course of the operation. The visualization helped in tumor localization and planning of the optimal access to the pathology. The whole flow of the performed procedures is presented in Fig. 1.

Additionally, due to the pandemic situation, the remote connection with the team of specialists was tested. The operator had the head-mounted display on his head and the team could see the operating room, the patient and the holograms fully remotely. It was possible to check and exchange opinions as well as interact with the data using special remote synchronization of the holograms.

### CarnaLife Holo system

CarnaLife Holo is a software that enables 3D holographic visualization of DICOM data that has been specifically designed for support of the mixed reality in HoloLens 1 and HoloLens 2. The system can work directly with the hospital PACS system for instantaneous imaging data retrieval in the operating room and the preoperative environment. Its main purpose is to provide diagnostic images to support the process of operation planning and also as a decision support tool during the operation. The system gives better spatial awareness to the user, enables interaction with the visualized patients data by using voice commands and hand gestures without breaking the sterile field. The images are synchronized between the workstation and the glasses and as a result, it is possible for the surgeon to adjust the images and also the radiologist can do that from the workstation in real-time. What is important, the hologram is created for the specific patient and can be individually adjusted by the operating surgeon or the cooperating specialist (radiologist, biomedical engineer). This ensures the best quality of the hologram for the support of the medical procedures and enhancement of the important structures in the patients' data.

The usage of the holographic visualization is not interfering with the current methods as it is used as a diagnostic and treatment support tool. The information gathered during the performed procedures confirms that the holograms do not interfere with the treatment as the hologram can be present only for the crucial times of the procedure. The weight of the head-mounted display is also negligible as the set fits the head tightly.

### IRE

Irreversible electroporation (IRE) is used as cytoreductive modality for patients with unresectable solid tumors. This is a technique using non-thermal electrical energy. It uses high-voltage, low-energy DC current up to 3 kV pulses. The electrical energy disrupts the cellular membrane integrity, causes loss of cellular homeostasis and ultimately results in cell death through both apoptosis and necrosis (Lee et al. 2010). IRE can be conducted in a surgical (open and laparoscopic) or percutaneous setting. The high-voltage electrical pulses are administered through 2 or more needle electrodes, forming electrical fields between each electrode pair. The needles are precisely placed in and around the tumor including a tumor-free margin. It preserves the vulnerable structures, including the major biliary and hepatic structures, large vessels, and the intestines (Vogel et al. 2016). In liver cancer, IRE was shown to be efficacious with low levels of local recurrences and only minimal complications. In pancreatic cancer, it proved to have significant survival benefit (Kourounis et al. 2017).

### MWA

Microwave ablation (MWA) destroys solid tumors using heat generated by microwave energy by using devices with frequencies at 900–2500 MHz. Solid organs and tumors with a high percentage of water are most conducive to this type of heating. The electromagnetic field works on two mechanisms: by agitating water molecules in the tissue causing friction and heat, and by displacement of ions leading to a collision with other ions, which converts kinetic energy into heat. The overall objective of thermal ablation removes the tumor and a 5–10-mm thick margin of seemingly normal tissue (Simon et al. 2005). The technique allows for flexible approaches to treatment, including percutaneous, laparoscopic, and open surgical access.

## Results

**Fig. 2:**
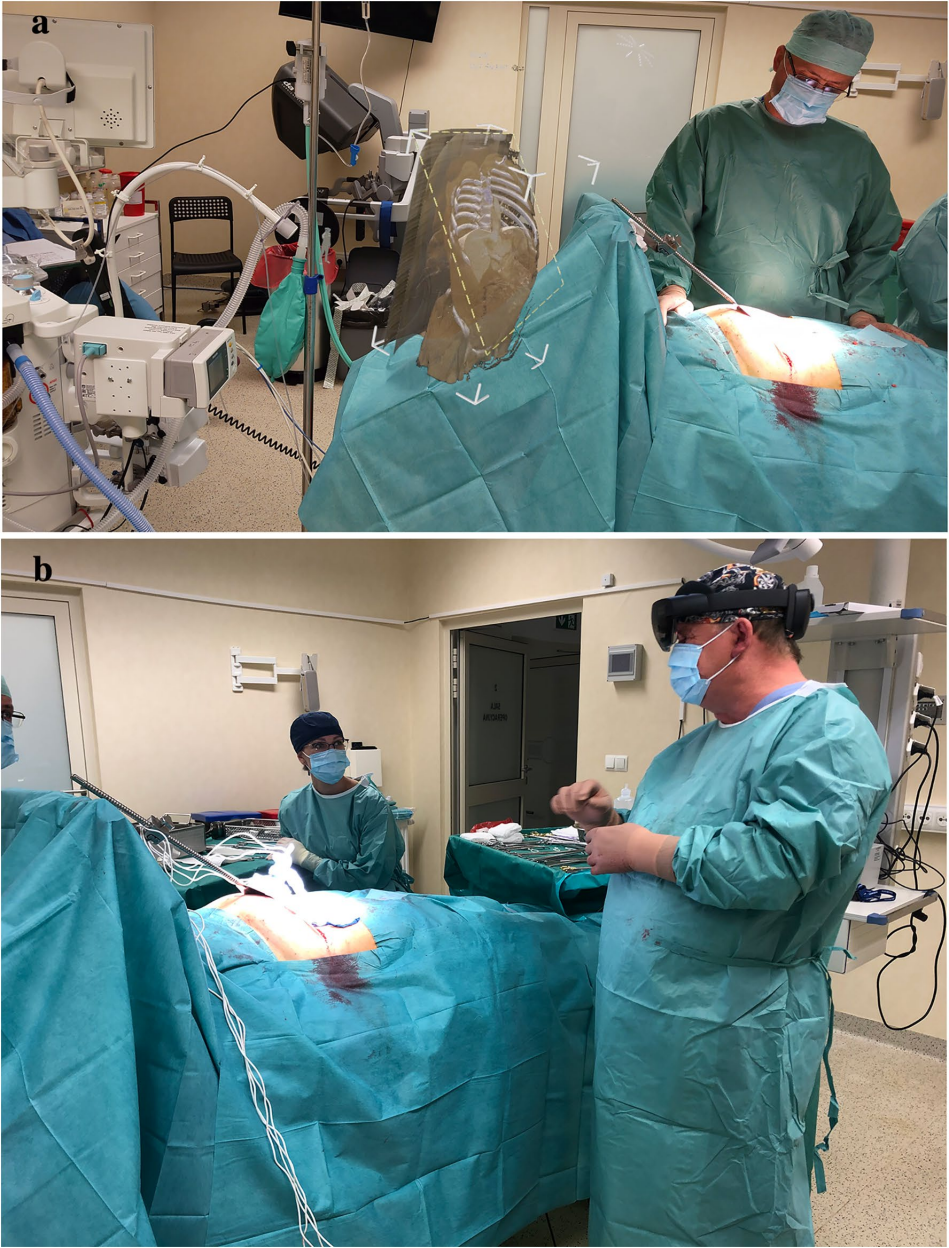
Hologram preview during the operation

The intraoperative hologram contributed to a better presentation of tumor size and locations. More precise setting of needles used to irreversible electroporation and for determining ablation line in case of liver metastases. In other cases, the hologram enabled localization and a safe approach for cholangiocellular carcinoma situated in a hilum. Surgeons could easily compare the real patient's anatomy to holographic visualization just before the operations. An exemplary image with the hologram is presented in Fig. 2. The use of minimally invasive surgical techniques in combination with CarnaLifeHolo resulted in the reduction of the procedure time by 1/3 compared to the time of surgery without the use of the CarnaLife Holo system, the mean was 62.5 min with the SD of 21.2 min and the associated period of general anesthesia, the mean was 85 min with the SD of 26.97 min. The period of hospitalization was short, the mean was 5.5 day with the SD of 1.15 days. The patients did not require analgesic treatment during hospital recovery. Oral food intake was introduced on the first postoperative day. Routine anticoagulation for Venous Thromboembolism prevention by using postoperative enoxaparin was included. There were no perioperative complications. The average period from discharge from hospital to the commencement of chemotherapy was 14 days, which is an excellent result correlated with the quality of life after the surgery. We did not observe any most common complications of our approach such as bleeding, infection, complications from general anesthesia, inflammation of the abdominal wall, blood clots.

As of the 19th May 2021, no patient’s deaths nor any complications were recorded in the group treated using the combination of MIS and CarnaLifeHolo. Every patient is being monitored and undergo systematic treatment in oncological centers.

## Discussion

Minimally invasive surgical procedures are defined as one of the safest and are associated with lower postoperative morbidity of a patient compared to a conventional approach for the same operation. These techniques need to apply a multidisciplinary approach to fulfill their potential. Forming a coherent team of specialists from various disciplines working in cooperation is crucial to obtain the best results. The main factors of the operation are a combination of the technological advances and the skills of the operator.

Mixed reality is a promising tool for support of surgical procedures. It can influence the time of the procedures by lowering the average time of the operation thanks to the better spatial awareness in the 3D medical imaging data like CT or MRI and also by the better preparation for the procedure during the preprocedural planning. The precise visualization facilitates the decisions about the incisions and the placement of the needles. The voice commands and hand gestures make it easier to adjust the holograms for personal needs without breaking the sterile field. It is also possible to pan through the hologram with a cutting plane that shows the inner parts of the imaging data in an interactive and immersive way.

During the tests, both HoloLens 1 and HoloLens 2 were checked for usefulness in the work of a surgeon. It turned out that HoloLens 2 is a much better match for applications in medicine especially thanks to the more ergonomic design. HoloLens 1 places a heavyweight on the nose of the user. In contrast, HoloLens 2 does not have any contact with the nose. HoloLens 2 had better image quality which resulted in easier tumor localization. The more precise depth cameras made it easier to interact with the hologram in a more natural and intuitive way. The field of view (FOV) in the HoloLens 2 is 52° and that gives much more freedom in looking at the holograms than in the predecessor that had the FOV of 30°. It all translated into higher satisfaction of the surgeon during the preoperative planning and also during the operation when using HoloLens 2 when it was compared to the HoloLens 1.

**Fig. 3:**
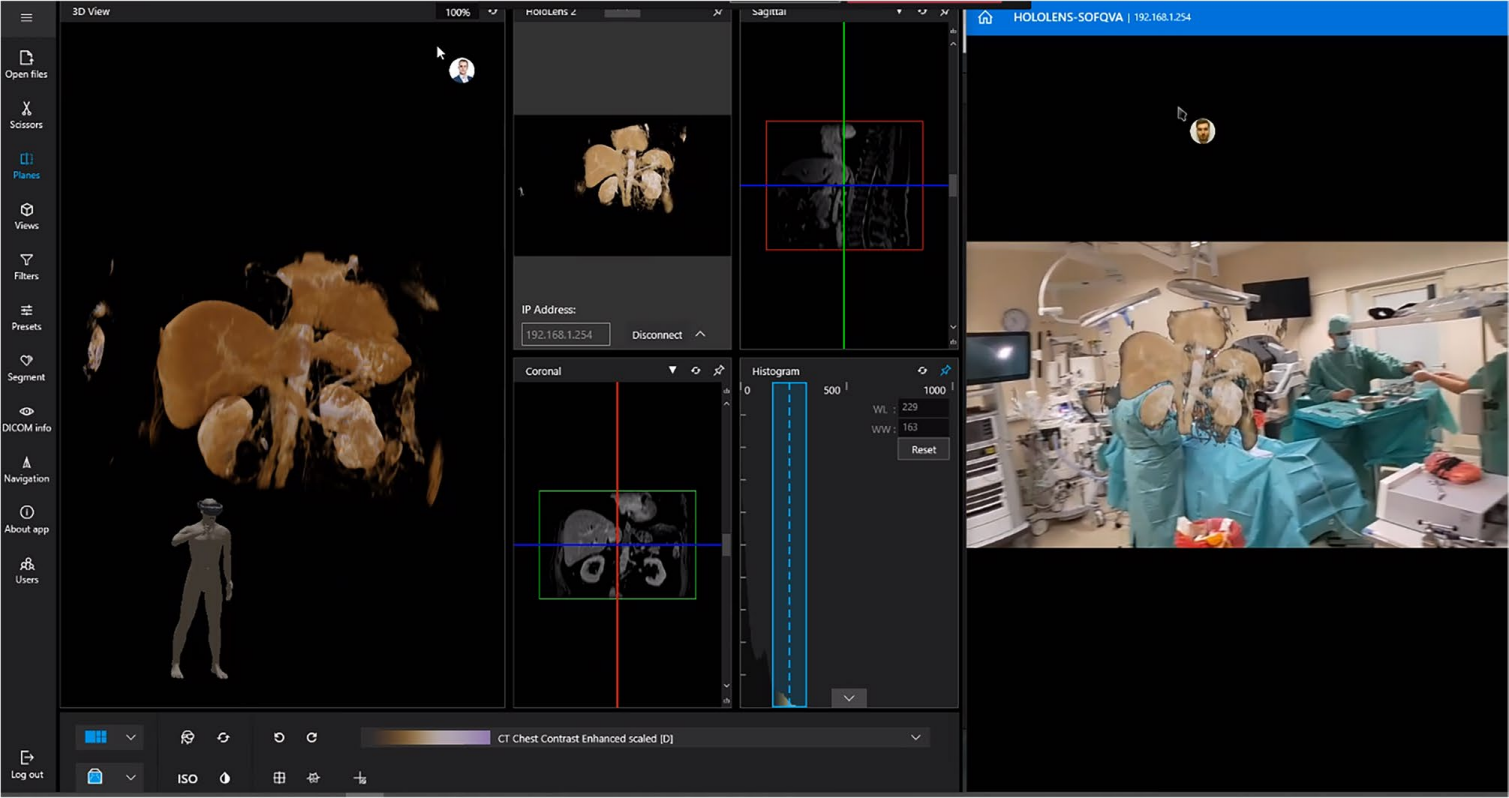
Remote connection view

In the preoperative planning, the multidisciplinary team prepared the visualization of medical data on the basis of computed tomography (CT) and/or magnetic resonance imaging (MRI). The consultation with the team can be done by using CarnaLife Holo both in the hospital or remotely. The exemplary view from the remote side is presented in Fig. 3.

The holographic visualization can be easily transferred to different cases in the oncological procedures but also in any other different area where the information about the depth and structures anatomy is important. In our opinion, this will find an application in any clinical routine where it is necessary for the assessment of complex structures like pulmonary arteries or bone fractures.

Based on the data from the performed procedures we predict our approach to improve equivalent oncologic or functional outcomes while decreasing perioperative complications and reducing hospital stay time, analgesic pain treatment and recovery time.

## Summary

In all presented cases, in the preoperative planning, the multidisciplinary team prepared the visualization of medical data on the basis of computed tomography (CT) and/or magnetic resonance imaging (MRI). It was used for planning the scope of the surgery and was the basis for the decision regarding the choice of the surgical technique. On the day preceding the operation, a visualization of the CT/MRI and planning of the procedure was done in mixed reality using the CarnaLife Holo system in a team of oncological surgeon and a 3D mixed reality engineer. Preoperative CT or MRI data were transformed into 3D holograms and displayed thanks to the head-mounted display (HMD). During operation, the surgeon’s field of view was augmented with a 3D- model of the patient’s relevant structures displayed in the mixed reality using the HMD.

The use of the CarnaLife Holo system made it possible to personalize the treatment and to select the proper surgical method. The intraoperative hologram contributed to better presentation of tumor size and locations, more precise setting of needles used to irreversible electroporation and for determining the ablation line in case of liver metastases, supported the use of minimally invasive surgery techniques.

Good performance status after surgery and the possibility of early initiation of chemotherapy significantly influences and determines the patient's prognosis. The combination of engineering—3D mixed reality (MR) techniques using CarnaLife Holo visualization system with innovative, minimally invasive surgery (IRA, MWA) for gastrointestinal tumors and systemic treatment (chemotherapy) might be a new way in personalized treatment of advanced cancers especially so far qualified as palliative.
